# Maturation of social attribution skills in typically developing children: an investigation using the social attribution task

**DOI:** 10.1186/1744-9081-6-10

**Published:** 2010-02-03

**Authors:** Zhouyi Hu, Raymond CK Chan, Grainne M McAlonan

**Affiliations:** 1 Neuropsychology and Applied Cognitive Neuroscience Laboratory, Department of Psychology, Sun Yat-Sen University, Guangzhou, China; 2 Department of Pharmacy, ZheJiang Pharmaceutical College, Ninbo, China; 3 Neuropsychology and Applied Cognitive Neuroscience Laboratory, Institute of Psychology, Chinese Academy of Sciences, Beijing, China; 4 Key Laboratory of Mental Health, Institute of Psychology, Chinese Academy of Sciences, Beijing, China; 5 State Key Laboratory for Brain and Cognitive Sciences, University of Hong Kong, Hong Kong Special Administrative Region, China; 6 Department of Psychiatry, the University of Hong Kong, Hong Kong Special Administrative Region, China

## Abstract

**Background:**

The assessment of social attribution skills in children can potentially identify and quantify developmental difficulties related to autism spectrum disorders and related conditions. However, relatively little is known about how these skills develop in typically developing children. Therefore the present study aimed to map the trajectory of social attribution skill acquisition in typically developing children from a young age.

**Methods:**

In the conventional social attribution task (SAT) participants ascribe feelings to moving shapes and describe their interaction in social terms. However, this format requires that participants understand both, that an inanimate shape is symbolic, and that its action is social in nature. This may be challenging for young children, and may be a potential confounder in studies of children with developmental disorders. Therefore we developed a modified SAT (mSAT) using animate figures (e.g. animals) to simplify the task. We used the SAT and mSAT to examine social attribution skill development in 154 healthy children (76 boys, 78 girls), ranging in age from 6 to 13 years and investigated the relationship between social attribution ability and executive function.

**Results:**

The mSAT revealed a steady improvement in social attribution skills from the age of 6 years, and a significant advantage for girls compared to boys. In contrast, children under the age of 9 years performed at baseline on the conventional format and there were no gender differences apparent. Performance on neither task correlated with executive function after controlling for age and verbal IQ, suggesting that social attribution ability is independent of cognitive functioning. The present findings indicate that the mSAT is a sensitive measure of social attribution skills from a young age. This should be carefully considered when choosing assessments for young children and those with developmental disorders.

## Background

The ability to understand the state of mind and intentions of other people is a critical faculty of man. It helps predict the behavior of others and is essential for appropriate social interaction. Conventional 'Theory of Mind' (ToM) tests of this skill have been criticized because they may rely too heavily on cognitive ability and fail to capture social skills needed in more 'naturalistic' conditions [1]. The 'Social Attribution Task' (SAT) is an adept measure of social skills which does not have complex reasoning demands [1,2]. In the original paradigm participants are shown a movie of 2 triangles and a circle moving around and in and out of a rectangle. These 2D shapes do not resemble people but typically developing individuals tend to spontaneously describe the actions in anthropomorphic terms endowing them social intentions, emotions, and personalities. Descriptions of relationships between the shapes are offered (e.g. friend), their actions are considered to have social intent (e.g., trapping, protecting), and the mental states of the various shapes are suggested. How a participant responds in the SAT provides a window onto their ability to ascribe social meaning to behavior. Klin extended the use of the SAT to uncover theory of mind limitations in people with autism who performed well on 'false belief' ToM tasks [1]. Subsequently the SAT has been adopted as a useful measure of the ability to infer beliefs and intentions in autism [3,4].

The ability to derive social meaning from visual stimuli is thought to develop spontaneously from early infancy. As the experience of social interaction increases social attribution skills become increasingly honed. Six to 8 year old children perform inconsistently when describing the SAT, and Thommen and colleagues found that children create ever more ambitious plots from age 7 [5-7]. Together studies suggest social attribution ability may not fully develop before the age of 8 years old. Thus, given that even typically developing children from younger age groups do not perform the SAT as well as older children or adults, using the task to study young participants with autism or other developmental disorder is potentially problematic [5].

The actual developmental trajectory of social attribution skills has not been systematically examined and is the focus of the present study. Given concerns regarding the appropriateness of the conventional SAT to younger children, we designed a modification of the SAT (mSAT) with animate rather than ambiguous stimuli. These stimuli should more readily be expected to interact in a social manner and therefore we predicted the task would be easier for younger children. The developmental trajectory of social attribution skills was then assessed using this version of the SAT in parallel with the conventional task.

The extent to which the development of social skills is part and parcel of more general cognitive maturation is debated. Mentalizing ability has been reported to correlate with executive function, especially tasks involving inhibitory control, even after controlling for age, gender, and verbal ability [8,9]. However, other evidence suggests that ToM is a discrete dimension, dissociable from other cognitive functions, and one which matures relatively independently from other skills [10]. For example, individuals with high functioning autism have marked difficulties with social interaction but relative sparing of other cognitive faculties [10-13]. Conversely, ToM is selectively spared relative to a general cognitive impairment in people with neuro-developmental disorders such as Down's and Williams' syndromes. This double dissociation between ToM and other high-level cognitive skills is taken as evidence that ToM is indeed a distinct, domain-specific skill. Therefore in this study we also examined the relationship between executive function and social attribution skills measured in both versions of the SAT.

## Methods

### Pilot study

The materials in the modified version of SAT (mSAT) were five cartoons including 'bridge', 'small dog', 'pig, wolf, and fox'. These were designed using the Macromedia Director MX 2004 program to have an enriched social content and to capture social situations that might be encountered in everyday life. The aim was to maximize younger viewers' tendency to recognize socially expressive actions and reactions. The basic procedures followed for the mSAT were the same as those described for the SAT by Klin [1,14]. Participants were shown the cartoons twice and asked to talk about the content. The first account was unprompted. In the second account, the experimenter promoted the participant to see the stimuli as characters. In the final account 3 parts of the cartoon were shown again and specific questions were asked. For example questions asked included the name of the stimulus (e.g. a dog), and a description of the action (e.g. helping). Finally the children were asked why they thought an interaction was occurring between 2 characters. Coding followed the protocol given in Klin [1,14]. In brief, Pertinence Index, was the proportion of total propositions which were vague and/or misattributed and/or irrelevant and/or inconsistent. The Salience Index, was the proportion of highly salient attributions made. The Theory of Mind Index: Cognition, was the proportion of total propositions which had mental state terms. The Theory of Mind Index: Affective, was the proportion of total propositions which contained emotion terms. The Animation Index, coded the overall quality of social attribution ability. The Person Index, coded the extent to which physical, behavioral, relationship or psychological details were used when asked what sort of 'people' the stimuli were. The Problem solving Index, was the proportion of correct answers given to direct questions about cartoons.

### Inter-rater reliability

Two independent and experienced raters scored responses blindly and in random order based on the 8 indices as defined in the conventional SAT [1]. The range of inter-rater reliability (Kappa agreement) for the modified SAT and SAT was 0.7 - 0.94 and 0.71 - 0.94, respectively, calculated from a random selection of 20 children (10 boys, 10 girls) aged 6 years (mean IQ = 99.80, *SD *= 14.06).

### Test-retest reliability

Test-retest reliability of mSAT and SAT was carried out using data from the same 20 children over a 4 week period. The test-retest reliability (Pearson r) for mSAT indices were all p < 0.01: Propositions (*r *= .89), Pertinence (*r *= .88), Salience Index (*r *= .90), ToM-Cognitive Index (*r *= .86), ToM-Affective Index (*r *= .84), Animation Index (*r *= .87), Person Index (*r *= .89) and Problem Solving (*r *= .88). The test-retest reliabilities (Pearson r) for SAT indices were all p < 0.05: Propositions Index (*r *= .85), Pertinence Index (*r *= .87), Salience Index (*r *= .82), ToM-Cognitive Index (*r *= .88), ToM-Affective Index (*r *= .73), Animation Index (*r *= .79), Person Index (*r *= .80) and Problem-Solving Index (*r *= .76).

### Concurrent validity

The concurrent validity for each index of the mSAT and SAT was calculated in the oldest group of children and showed that for this age group the 2 versions characterized social attribution skill similarly. Propositions (*r *= .87, *p *< .001), Pertinence (*r *= .80, *p *< .001), Salience Index (*r *= .78, *p *< .001), ToM-Cognitive Index (*r *= .83, *p *< .001), ToM-Affective Index (*r *= .91, *p *< .0017), Animation Index (*r *= .93, *p *< .001), Person Index (*r *= .90, *p *< .001), and Problem Solving (*r *= .92, *p *< .001).

### Study of age-related development of social attribution skill

#### Participants

A sample of 154 children (76 boys, 78 girls) was recruited from regional primary and middle schools in Guangzhou, China. The mean age of the total sample was 9.88 years (SD = 2.30) and the mean IQ was 106.67 (SD = 13.98). All participants were right-handed and no behavioral or academic difficulties had been recorded in their annual school or parental reports. Exclusion criteria were a diagnosed developmental disorder (e.g., autism, ADHD), major medical disorder (e.g., epilepsy), or regular medication. Teachers invited them to participate and their parents give consent for participation in the present study, as approved by the local institutional review board. Participants were made aware that their responses would be recorded in writing and audio-taped and all responses were recorded verbatim. They were divided into eight year groups from 6 - 13 years old but tested in random order. Descriptive statistics for each age group are given in Table 1.

**Table 1:** Demographic and Cognitive Characteristics by all age groups (mean; SD)

Group (boys: girls)	6-yrs (10:10)	7-yrs (10:10)	8-yrs (10:11)	9-yrs (10:9)	10-yrs (10:10)	11-yrs (11:9)	12-yrs (9:10)	13-yrs (6:9)
VIQ	96.00 (10.74)	98.65 (7.68)	103.48 (13.17)	109.74 (15.56)	112.05 (13.79)	105.10 (13.86)	114.00 (13.33)	112.73 (11.12)
PIQ	110.25 (20.42)	105.50 (15.71)	106.90 (17.04)	116.74 (12.59)	115.65 (18.09)	104.95 (28.33)		
FSIQ	99.80 (14.06)	102.70 (10.41)	104.71 (12.96)	113.58 (14.04)	115.45 (15.58)	107.95 (15.36)		

### Materials

#### General cognitive ability and executive function testing

The short form of the Chinese version of the Wechsler Intelligence Scale for children -- Revised (WISC-R) was used to assess general cognitive ability [15]. Tests of verbal ability included arithmetic, vocabulary, and digit span; tests of performance ability included block design and object assembly. An Executive function battery incorporated the following conventional tests following standard protocols:

Cognitive flexibility assessed using the modified version of WCST; Inhibitory control measured with the Chinese version of the Stroop test (Victoria version, adapted Chinese version) and Verbal fluency quantified by asking the participants to name as many animals as possible in 1 minute [16,17].

#### Procedure

The SAT, mSAT and cognitive test battery were administered in random order to each child in 3 × 20 min sessions held over 1 week.

### Data analysis

SAT and mSAT scores were analysed in two-way analyses of variance (ANOVA) with age and gender (8 × 2) as between-subjects variables. Planned comparisons and/or post hoc comparisons with the Newman-Keuls test with Bonferroni corrections were conducted to further define any significant effect of age, gender or interaction (significance was assumed at a value of *p *< .05). The relationship between test variables was estimated using Kendall's rank correlation coefficients and partial correlations, depending on the distribution of the data. All analyses were performed on SPSS 13.0.

## Results

**Figure 1:**
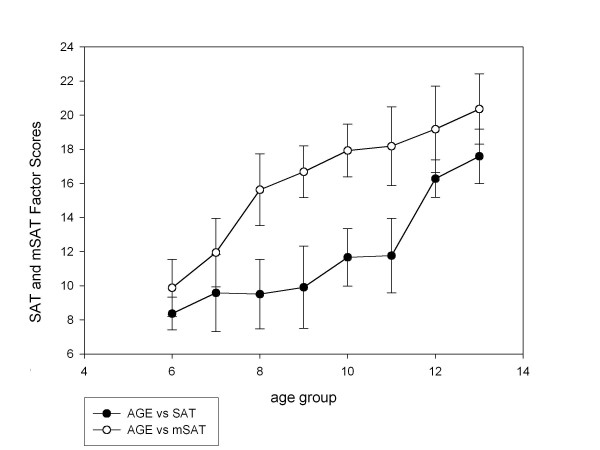
**The development pattern of SAT and mSAT indices**.

Table 2 and Table 3 list means and standard deviations for each age level on the SAT and mSAT indices. Table 4 presents main effects in 8 age groups, gender, and the age (8) × gender (2) interaction among age groups of SAT and mSAT indices. Figure 1 displays a summation of all indices in both tasks across the age groups studied. It illustrates that, although both versions captured a significant developmental improvement in social attribution skills [*F*(7,146) = 57.496, *p *= 0.0005 for original SAT; *F*(7,146) = 62.286, *p *= 0.0005 for mSAT], a floor effect in younger age groups (6 - 9 years) was clearly evident in the SAT data. Performance on SAT and mSAT did not correlate with verbal IQ. In general, girls out-performed boys on a number of SAT and mSAT indices including ToM.

**Table 2:** Performance on SAT measures among 8 age groups (mean; SD)

SAT	6-yrs	7-yrs	8-yrs	9-yrs	10-yrs	11-yrs	12-yrs	13-yrs
Proposition	5.75 (.79)	5.90 (1.25)	6.05 (.97)	6.32 (1.53)	7.15 (1.35)	7.25 (1.29)	8.63 (1.12)	9.00 (.85)
Pertinence	.06 (.10)	.04 (.08)	.03 (.08)	.03 (.07)	.03 (.06)	.04 (.08)	.00 (.00)	.00 (.00)
Salience	.27 (.05)	.28 (.07)	.29 (.06)	.31 (.08)	.35 (.07)	.35 (.07)	.43 (.06)	.45 (.04)
ToM Cognitive	.05 (.08)	.06 (.10)	.10 (.13)	.05 (.09)	.07 (.11)	.09 (.11)	.08 (.10)	.10 (.09)
ToM Affective	.00 (.00)	.03 (.06)	.02 (.06)	.02 (.06)	.04 (.08)	.03 (.08)	.06 (.09)	.05 (.08)
Animation	1.00 (.65)	1.40 (.50)	1.43 (.68)	1.37 (.50)	1.60 (.88)	1.70 (.73)	3.05 (.52)	3.13 (.52)
Person	.90 (.31)	1.50 (.89)	1.19 (.81)	1.47 (.96)	1.95 (.94)	1.90 (.79)	3.32 (.58)	4.00 (.53)
Problem-solving	.34 (.12)	.37 (.11)	.38 (.17)	.34 (.11)	.48 (.16)	.41 (.13)	.72 (.18)	.85 (.15)

**Table 3:** Performance on mSAT measures among 8 age groups (mean; SD)

mSAT	6-yrs	7-yrs	8-yrs	9-yrs	10-yrs	11-yrs	12-yrs	13-yrs
Proposition	6.60 (1.19)	7.00 (.97)	8.14 (1.31)	8.89 (.94)	9.50 (1.15)	9.65 (1.53)	10.11 (1.70)	10.67 (1.35)
Pertinence	.06 (.11)	.02 (.05)	.06 (.09)	.04 (.08)	.04 (.09)	.03 (.05)	.00 (.00)	.00 (.00)
Salience	.42 (.10)	.46 (.07)	.51 (.11)	.57 (.08)	.61 (.09)	.63 (.10)	.67 (.11)	.71 (.09)
ToM Cognitive	.16 (.08)	.23 (.13)	.27 (.11)	(.11)	.29 (.11)	.28 (.11)	.35 (.13)	.41 (.07)
ToM Affective	.06 (.08)	.07 (.09)	.17 (.08)	.17 (.09)	.18 (.08)	.21 (.11)	.21 (.10)	.23 (.08)
Animation	1.60 (.50)	1.95 (.76)	2.90 (.54)	3.00 (.47)	3.30 (.57)	3.25 (.79)	3.32 (.82)	3.53 (.92)
Person	.65 (.81)	1.60 (.88)	2.76 (.77)	2.95 (.78)	3.15 (.59)	3.30 (.80)	3.63 (.50)	3.93 (.59)
Problem-solving	.34 (.12)	.62 (.15)	.80 (.17)	.78 (.19)	.86 (.19)	.84 (.19)	.89 (.15)	.88 (.16)

**Table 4:** Main effects in 8 groups of age, gender, and the Age × Gender interaction among age groups of SAT and mSAT Indices

SAT Index	Age			Gender		Interaction	
	
	*F*	*p*	Post Hoc Significant differences	*F*	*p*	*F*	*p*
Proposition	**19.352**	**.0005**	6-9&10-11&12-13	3.625	.059	.767	.616
Pertinence	1.734	.106	No differences	2.432	.121	**2.337**	**.028**
Salience	**20.471**	**.0005**	6-9&10-11&12-13	**5.469**	**.021**	1.140	.342
ToM Cognitive	.776	.608	No differences	1.630	.204	.367	.920
ToM Affective	1.354	.230	No differences	.015	.902	.702	.670
Animation	**26.199**	**.0005**	6-9&10-11&12-13	1.181	.279	.581	.770
Person	**34.099**	**.0005**	6&7-9&10-11&12&13	2.116	.148	1.249	.281
Problem-Solving	**30.121**	**.0005**	6-11&12&13	**5.573**	**.020**	1.425	.200
mSAT Index							
Proposition	**23.422**	**.0005**	6-7&8-9&10-11&12-13	2.399	.124	.534	.808
Pertinence	1.925	.070	No differences	.217	.642	1.000	.434
Salience	**21.260**	**.0005**	6-7&8-9&10-11&12-13	2.048	.155	.760	.622
ToM Cognitive	**8.374**	**.0005**	6&7-11&12-13	**11.598**	**.001**	1.755	.101
ToM Affective	**10.214**	**.0005**	6-7&8-13	**17.190**	**.0005**	1.253	.278
Animation	**22.738**	**.0005**	6-7&8-9&10-13	**7.697**	**.006**	**3.611**	**.001**
Person	**46.059**	**.0005**	6&7&8-11&12-13	.399	.529	**3.061**	**.005**
Problem-Solving	**25.880**	**.0005**	6&7&8-13	**8.604**	**.004**	.914	.498

Partial correlation analysis controlling for age and verbal IQ was conducted to explore the relationships between social attribution ability and executive function. The results showed scores from executive function measures were generally not related to the SAT and mSAT indices score after controlling for age and verbal IQ (Table 5). The exceptions were significant correlations between the Problem-Solving Index and the number of categories of WCST completed (*r *= .225, *p *= .014); the Proposition Index and verbal fluency (*r *= .311, *p *= .001), and the Salience Index and verbal fluency (*r *= .240, *p *= .009).

**Table 5:** Partial correlation analyses between SAT and mSAT Indices and Executive Functions in healthy school-aged children, controlled for age and VIQ (N = 120)

SAT	Proposition	Pertinence	Salience	ToM-C	ToM-A	Animation	Person	Problem-solving
Stroop-RT	.051	.030	.051	.157	.099	.070	.063	.019
Stroop-ERR	.036	-.014	.040	.028	.048	.090	.060	-.049
WCST-CA	.111	-.001	.089	.059	.085	.035	.074	.225*
WCST-PR	-.023	.014	-.033	.114	-.044	.045	.037	.053
WCST-PE	.008	.045	.029	.113	.059	.058	.067	.086
VF	.001	.078	.041	.080	.037	.073	.014	.052
**mSAT**	**Proposition**	**Pertinence**	**Salience**	**ToM-C**	**ToM-A**	**Animation**	**Person**	**Problem** **-solving**
Stroop-RT	-.007	.100	-.038	.064	.007	-.029	.044	-.002
Stroop-ERR	-.007	-.023	-.001	-.049	.006	-.055	-.004	.119
WCST-CA	-.125	.145	-.174	-.085	-.035	.067	-.117	.009
WCST-PR	.058	-.131	.108	-.042	.034	-.019	-.029	.013
WCST-PE	.058	-.126	.105	-.071	.020	-.034	-.031	-.011
VF	.311***	.020	.240**	-.036	-.064	.100	-.061	.017

Stroop-RT: Reaction Time Stroop word-color interference. Stroop-ERR: Errors Stroop word-color interference. WCST-CA: Categories completed Wisconsin Card Sorting Test. WCST-PR: Perseverative responses Wisconsin Card Sorting Test. WCST-PE: Perseverative errors Wisconsin Card Sorting Test. VFT: Verbal Fluency.

## Discussion

The main findings in this study are summarized below:

1. The mSAT showed a steady improvement in social attribution skills with age (Figure 1). However, the conventional SAT showed a floor effect between the ages of 6 and 9 years and a year on year improvement thereafter.

2. Girls outperformed boys in half of the parameters in the mSAT.

3. Social attribution ability was not linked to executive function in typically developing children.

### The developmental pattern of social attribution in school-aged children

One of the primary purposes of this study was to characterize the developmental progression of social attribution ability in healthy school-aged children. As expected, the present findings showed social attribution skills advance with age in this sample. However, the conventional SAT was insensitive to changes between the ages of 6 and 9 years, while the mSAT picked up the improvements made in these younger age groups.

The results of performance on the Salience Index in both versions of the SAT strongly indicate that the mSAT is easier for younger children. In the conventional SAT, younger children described around 27% of the social events, while older children described 45%. This contrasts the mSAT in which younger children found 42% of the social elements, and older children 71%. Thus it seems that young children do not tend to readily attribute the ambiguous geometric stimuli in the conventional task with social meaning. Conversely the stimuli in the mSAT may be more accessible for social evaluation in this age group.

Although the mSAT appeared to be simpler for younger age groups, the task still elicited a smaller repertoire of mental state terms from young children compared to older children. The conventional SAT also captured fewer mental state attributions in younger age groups, but showed minimal increase in these indices with age (4% to 10% for ToM-Cognitve index and 0% to 5% for ToM-Affective index). Importantly, the mSAT was sensitive to age-related changes in mentalizing ability, revealing a significant increase in ToM skill with age (16% to 41% for ToM-Cognitive index and 4% to 23% for ToM-Affective index). This latter indicates the mSAT may have an advantage when assessing possible developmental delay in mentalizing ability in young children compared to the conventional SAT.

Consistent with a general pattern of improvement in social skills with age, both the mSAT and SAT results showed older children scored significantly higher on the Animation index, a measure of the "capacity" for social attribution [1]. Similarly older children scored significantly higher on the Person index reflecting their greater appreciation of the complexities of personality. Younger children were constrained to personal judgments involving simple size and shape attributes. In addition, a better grasp of social situations in older children compared to younger children was documented by their accuracy in Problem solving in both SAT ratings (85% for SAT and 88% for mSAT).

Taken together, the results point to important differences in social attribution skills of younger children compared with older children. Up to 7 years old, children preferentially explain events in physical terms even in the mSAT cartoon (e.g., going for a ride then feeding her horse hay). During middle childhood (8-10 years), children begin to consider the intentions behind the motion on screen (e.g., running away from a bully because he knew he'd take his lunch). By the age of 11 years, the mental states of the characters were a core component of the monologues and often drew upon personal experience and a rich understanding of human traits (e.g., being afraid to 'speak up' because she learned the hard way that it didn't pay). It seems the acquisition of social attribution skills progresses through an early grasp that physical actions have outcomes. In older children these actions can be more readily understood within a social framework with psychological consequences. A mature social attribution ability allows the oldest children to evaluate scenarios not just on the overt actions taking place, but also on the social interactions driven by the mental states of the characters involved.

### Gender difference in social attribution ability

In addition to the developmental pattern of social attribution ability, we also identified a gender effect on this ability. Girls tend to have a general developmental advantage over boys of the same age[18]. Consistent with this, we found that social attribution skills in girls were more advanced than boys on both tasks; girls out performed boys on half of the social attribution indices, on the other indices there was no effect of gender. There appears to be important gender differences in the way children approach social problems and solve interpersonal conflicts [19]. The literature suggests that girls are rather more socially adept than boys. They grasp the intentions of others more competently, and more easily formulate effective solutions to social problems [20]. However, few investigators have specifically examined gender differences in mentalizing and the present results indicate such gender differences in development of social skills deserve further attention.

### The relationship between social attribution ability and other cognitive abilities

A large number of studies have shown that ToM task performance is correlated with verbal ability [21-23]. The most straightforward explanation for this is that conventional ToM tasks are tested verbally. While it could be argued that verbal ability must also impact upon performance of SAT and mSAT, we found that after controlling for age, there was no relationship between SAT/mSAT and verbal IQ. In addition we observed no relationship between social attribution ability and executive function. This suggests that social attribution ability, at least as tested in the SAT, is a domain-specific ability that is independent of executive functioning [11]. This fits with evidence that mentalizing skills and executive function have distinct neural substrates [24,25]. However, it runs contrary to evidence from correlation studies that executive function and ToM are fundamentally linked in development [9].

One explanation for this discrepancy may be the extent to which more formal tests of mentalizing ability also tap on executive functions. Clearly successful reasoning about mental states could well demand some level of executive skill. For example, in the standard, unexpected-location false belief task, young children who have relatively poor inhibitory control could find it a challenge to resist pointing out the obvious location of the object in question[26]. The more naturalistic setting of the SAT and mSAT may be less demanding of executive resources.

### Limitations

Our assessment of executive functioning and social attribution correlations had a number of limitations. A more comprehensive executive function battery would better define any relationship between SAT performance and cognitive ability. For example, inclusion of more specific tasks dissecting only one component of executive functioning and linguistic ability, such as Hayling Sentence Completion (for semantic inhibition) might have been appropriate. Despite the fact that we controlled for verbal IQ and age, neither the SAT or mSAT should be considered completely independent of linguistic expertise since social attribution ability is scored from a verbal response. In future it would be useful to rise to the challenge of designing tasks to capture mentalizing skills in individuals with poor language. Such tasks would also help establish what role language plays in performance of able individuals with autism spectrum who do successfully pass standard ToM tasks [27]. It should also be remembered that our mapping of the maturation of social attribution skills is cross-sectional. We accept that longitudinal designs are a more valid and reliable means of assessing developmental change and will be required to verify our results. Further validation of the mSAT will be required in order to determine test-retest reliabilities and improve inter-rater reliabilities on its indices. Finally, the present findings are a record of behavioral observations, in future studies it would be interesting to employ concurrent functional neuroimaging techniques to track the development of the fundamental neural systems underpinning social ability.

## Conclusions

Our data indicates that social attribution ability improves with age. However, a modified version of the SAT was more sensitive to age-related changes in children between the ages of 6 - 9 years old. This important observation should be borne in mind when extending the study of social attribution to children with developmental disorders in this age group. Clearly a floor effect in typically developing children would mask any between group differences in social attribution. The mSAT described in the present study is available from the authors on request. We hope it can make a useful contribution to the assessment of children with social difficulties and potentially be applied to assess the utility of interventions aimed at improving theory of mind skills.

## List of abbreviations

ADHD: Attention Deficits and Hyperactivity Disorders; ANOVA: Analyses of variance; IQ: Intelligence quotient; mSAT: Modified Social attribution task; SAT: Social attribution task; SD: Standard deviation; ToM: Theory of Mind; WCST: Wisconsin Card Sorting Test; WISC-R: Wechsler Intelligence Scale for children -- Revised.

## Competing interests

The authors declare that they have no competing interests.

## Authors' contributions

ZH conceived and designed the study, administered the tests, analyzed data and wrote the manuscript. RCKC conceived and designed the study and wrote the manuscript. GMM wrote the manuscript. Each author read and approved the final version of the manuscript.
